# ‘A Constant Black Cloud’: The Emotional Impact of Informal Caregiving for Someone With a Lower-Grade Glioma

**DOI:** 10.1177/10497323231204740

**Published:** 2023-11-15

**Authors:** Ben Rimmer, Michelle Balla, Lizzie Dutton, Joanne Lewis, Richéal Burns, Pamela Gallagher, Sophie Williams, Vera Araújo-Soares, Tracy Finch, Linda Sharp

**Affiliations:** 1 Population Health Sciences Institute, 5994 Newcastle University, Newcastle University Centre for Cancer, Newcastle upon Tyne, England; 2 Faculty of Medical Sciences, 5994 Newcastle University, Newcastle upon Tyne, England; 3 5983 Newcastle Upon Tyne Hospitals NHS Foundation Trust, Newcastle upon Tyne, England; 4 364010 Faculty of Science, Atlantic Technological University, Sligo, Ireland; 5 Health and Biomedical Strategic Research Centre, 364010 Atlantic Technological University, Sligo, Ireland; 6 School of Psychology, 63856 Dublin City University, Dublin, Ireland; 7 Centre for Preventive Medicine and Digital Health, Department for Prevention of Cardiovascular and Metabolic Disease, Medical Faculty Mannheim, 639957 Heidelberg University, Heidelberg, Germany; 8 Department of Nursing, Midwifery and Health, 5995 Northumbria University, Newcastle upon Tyne, England

**Keywords:** emotional impact, lower-grade glioma, informal caregiving, qualitative

## Abstract

Those closest to people with lower-grade gliomas (LGGs) often assume the role of informal caregiver (IC). The additional responsibilities mean ICs of people with cancer can experience adverse impacts on their own lives. We explored the emotional impact of informal caregiving for people with LGGs. This was a descriptive qualitative study within the multi-method Ways Ahead project. We conducted semi-structured interviews with individuals from the United Kingdom, who currently, or in the past 5 years, informally cared for someone with an LGG. Interviews encompassed experiences of emotional impact as a consequence of caregiving for someone with an LGG. Inductive thematic analysis was undertaken. We interviewed 19 ICs (mean age 54.6 years; 14 females, 5 males). Participants reported substantial emotional impact. Four themes and associated subthemes were generated: *Emotional responses to the illness* (e.g. feeling helpless), *Emotional responses to the unknown* (e.g. anxiety about future uncertainty), *Emotional consequences of care recipient changes* (e.g. challenges of changed relationship dynamics), and *Emotional weight of the responsibility* (e.g. feeling burnout). Emotional impact in one area often exacerbated impact in another (e.g. future uncertainty impacted feelings of helplessness). Participants detailed the factors that helped them manage the emotional impact (e.g. being resilient). ICs of people with LGGs can experience wide-ranging emotional responses to and impacts of the illness, uncertain prognosis, care recipient changes, and the toll of caregiving. Adjustment and resilience are key protective factors, though further consideration of ways to identify and fulfil the emotional support needs of ICs of people with LGGs is required.

When an individual is diagnosed with cancer, those closest to them (e.g. family members, partners, and close friends) often assume the role of informal caregiver (IC), which includes additional practical (e.g. managing symptoms) and psychosocial (e.g. emotional support) responsibilities (Ullgren et al., 2018). The support provided by ICs is integral to the care recipient’s self-management of their condition (Schulman-Green et al., 2021). However, as a consequence of caregiving responsibilities, ICs can experience wide-ranging physical, psychological, relationship, and economic burden (Girgis et al., 2013; Tan et al., 2018; Unsar et al., 2021). A substantial proportion of ICs report unmet needs for support, the most prominent of which relate to psychological or emotional issues (Balfe et al., 2016; Wang et al., 2018).

Past research suggests that the nature of the caregiver burden may vary as the care recipient progresses through different phases of the cancer journey (e.g. increasing role demands at diagnosis and managing with fear of cancer recurrence in survivorship) (Northouse et al., 2012). In addition, ICs may experience distinct challenges, determined by the care recipient’s symptoms and impairments as a result of the cancer and its treatment, for example, dealing with strained relationships due to personality changes in people with brain tumours (Heckel et al., 2017; Ownsworth et al., 2015). It is, therefore, important to understand the experiences of ICs of people with different types of cancer to identify specific support needs.

Primary brain tumours are a heterogeneous collection of benign and malignant neoplasms arising in the brain and central nervous system. In 2020, worldwide, an estimated 300,000 new diagnoses of primary brain and central nervous system tumours were made (Sung et al., 2021). The most common malignant tumours of the brain are gliomas, which can be high or low grade (Miller et al., 2021). Lower-grade gliomas (LGGs) are a sub-group of malignant brain tumours largely diagnosed in adults in their 30s and 40s, at a crucial time in an individual’s work and family life (Bauchet, 2017). These tumours are mostly incurable, often progress to a high-grade glioma (HGG), and limit peoples’ life expectancy by, depending on the sub-type, 5–15 years (Bauchet, 2017; Dixit & Raizer, 2017).

Living long term with the impact on daily living (e.g. work, transport, and relationships) (Losing Myself | The Brain Tumour Charity, 2015) of diverse, often co-occurring, symptoms (e.g. fatigue and seizures) and impairments (e.g. cognitive deficits) (Rimmer et al., 2022) could have a profound impact not only on the person with an LGG but also on those closest to them. Since people with brain tumours frequently cite the importance to them of support from informal networks (Cornwell et al., 2012), caregiving burden needs to be better understood.

The few available studies of ICs of people with brain tumours show that greater perceived caregiving burden can exacerbate anxiety and depression in ICs (Zamanipoor Najafabadi et al., 2021), and vice versa (Q. Li et al., 2022), and that ICs face challenges with adjustment (e.g. acceptance of the diagnosis and prognosis, and negotiating changes in family roles) (Cavers et al., 2013), isolation, and subsequent loneliness (Kirby et al., 2022). Beyond this, the existing literature for caregiving burden in ICs of people with LGGs is limited, with studies typically including heterogeneous samples of LGG and HGG care recipients (Chen et al., 2021), groups which have very different prognoses (Pellerino et al., 2022). Moreover, available support services and interventions for ICs have largely been developed for ICs of people with HGGs (Heinsch et al., 2022) and lack continuity across the illness trajectory (Lion et al., 2023); thus, ICs of people with LGGs may have limited access to formal support. The nature of the prognosis, the impact of the tumour and its treatment, and limited available support services may mean, therefore, that ICs of people with LGGs face different challenges than ICs of people with HGGs or other cancers.

One existing qualitative study focused on LGG care recipients, underlining the emotional distress experienced by their next of kin (Edvardsson & Ahlström, 2008). The participant group averaged 12 years since the LGG diagnosis, providing long-term insight, congruent with the suggestion that ICs of people with LGGs may experience sustained high risk of depression (Boele et al., 2022). Thus, while psychological burden appears to be prominent in ICs of people with LGGs, more knowledge is needed to understand the emotional impact of caregiving responsibilities, living with care recipient changes, and how this is managed. This study, therefore, aimed to explore the emotional impact of being an IC for someone living with an LGG diagnosis.

## Method

### Design

This qualitative study – nested within the multi-method Ways Ahead project – had a descriptive design in recognition, and to facilitate exploration, of the subjective and diverse nature of participants’ experiences in an area where little is known (Doyle et al., 2020). We used semi-structured interviews to explore the impact and support needs of ICs involved in the support of people with LGGs. Ways Ahead (Rimmer et al., 2020) was reviewed and approved by the Wales Research Ethics Committee (REC ref.: 20/WA/0118).

### Participants and Recruitment

Participants were family members or friends of someone with an LGG (specifically, a grade 2 astrocytoma or grade 2 or 3 oligodendroglioma (Louis et al., 2021)) who lived in the United Kingdom. Individuals were eligible if they were aged 18 years or older and currently, or had in the past 5 years, informally cared for someone with an LGG; individuals who were a caregiver in the past 5 years, but who were bereaved at the time of recruitment, were therefore eligible.

Potentially eligible ICs were identified via three avenues: (1) a person with an LGG interviewed in another phase of the project was asked to nominate someone involved in their support and pass on a participant information sheet to the nominated IC; (2) health professionals at collaborating National Health Service (NHS) sites identified and provided eligible ICs with a participant information sheet; and (3) an advert with the participant information sheet attached was disseminated by the researcher (BR) through the Brain Tumour Charity’s networks. In all cases, ICs were approached as ‘family members or friends’ and asked to call or email the study team to register their interest. BR and LD called each interested IC to confirm eligibility, afford the opportunity to ask questions, and if the individual was eligible and willing, arrange a convenient interview date and time. Recruitment was conducted between August 2020 and March 2022.

### Data Collection

BR and LD, both trained and experienced in qualitative research, conducted the interviews. All interviews were undertaken remotely using video-conferencing software (e.g. Zoom or Microsoft Teams) or telephone, as per interviewee preference. Immediately prior to each interview, verbal, audio-recorded consent was acquired.

A topic guide (Supplementary file 1), comprising open questions informed by the literature and expert knowledge, was followed to semi-structure the interviews. The topic guide was modified prior to data collection following brain tumour public and patient involvement review; this included review by ICs. Throughout data collection, new issues raised in interviews were added to the topic guide, to be explored in subsequent interviews.

To begin, participants were invited to broadly reflect on their experiences of supporting someone living with an LGG. We then explored participants’ views on how the care recipient has been impacted by the tumour and its treatment, and their own impact and support needs across several areas (e.g. emotions, family, and transport). For each area, participants were asked about their caregiving responsibilities and what, and when, they received or needed (in)formal support. Probe questions were used, where appropriate, to further explore responses. Participants could also raise any additional issues they felt were important. Finally, a £20 voucher was offered to thank them for their time, as well as a debrief sheet with details of charities and helplines, should they have any questions or require support. Interviews were audio-recorded and lasted 85 minutes on average (range 54–110 minutes).

### Data Analysis

Participants were allocated a unique participant ID. Interviews were transcribed verbatim and anonymised by an external transcription service. For accuracy, transcripts were checked, by the interviewers, against the audio-recordings. All participants spoke very strongly about the emotional impact and their needs for emotional support; consequently, the present analysis aimed to provide a detailed exploration and understanding of the emotional impact of being an IC for someone with an LGG. An inductive thematic analysis was undertaken (Braun & Clarke, 2006; Clarke & Braun, 2021); this was chosen for its flexibility in being able to develop patterns of meaning, directed by the content across the IC dataset, to understand how the emotional impact of supporting someone with an LGG might be experienced in different ways.

Throughout data analysis, we took several steps to ensure rigour: (1) Analysis occurred in parallel with data collection to ensure that subsequent interviews explored any new issues raised. (2) Following independent familiarisation with the data, two trained researchers (BR and MB) generated initial codes for data which related to the emotional impact of caregiving within a sample of transcripts (*n* = 5 of 19). (3) Preliminary codes were discussed between the researchers to identify similarities and reach consensus on any differences, before suggesting potential themes at the semantic level. (4) Remaining transcripts were coded by BR and themes further developed. As analysis progressed, findings and uncertainties were regularly discussed with the wider analysis team (MB and LS) and themes were reviewed and refined accordingly. (5) Final themes and subthemes were defined and named and are reported with supporting quotes; quotes are accompanied by the IC’s age at interview and their relationship to the care recipient. (6) Recruitment ceased when reasonable data sufficiency occurred; this was determined by the perception that there was sufficient data to support and understand the emotional impact experienced by ICs (Low, 2019).

## Results

### Participant Characteristics

Twenty-four ICs registered their interest in the study. The tumour was not an LGG for two care recipients, so 22 were eligible. Nineteen ICs were interviewed, seven recruited through NHS sites and 12 through the Brain Tumour Charity. At the time of interview, mean age was 54.6 years (range 36–78 years). Fourteen participants were female and five were male. Eighteen participants were married and one was single. Fifteen participants were spouses, two sisters, and two mothers of the care recipients. Six participants (all spouses) had children (aged <18); three participants had one child and three had two children. Thirteen participants were working (10 in full-time and three in part-time employment), four had retired, and two were caring for family. Mean time in full-time education was 14.9 years (range 10–18 years). None of the participants were bereaved.

### Overview of Findings

**Figure 1.**
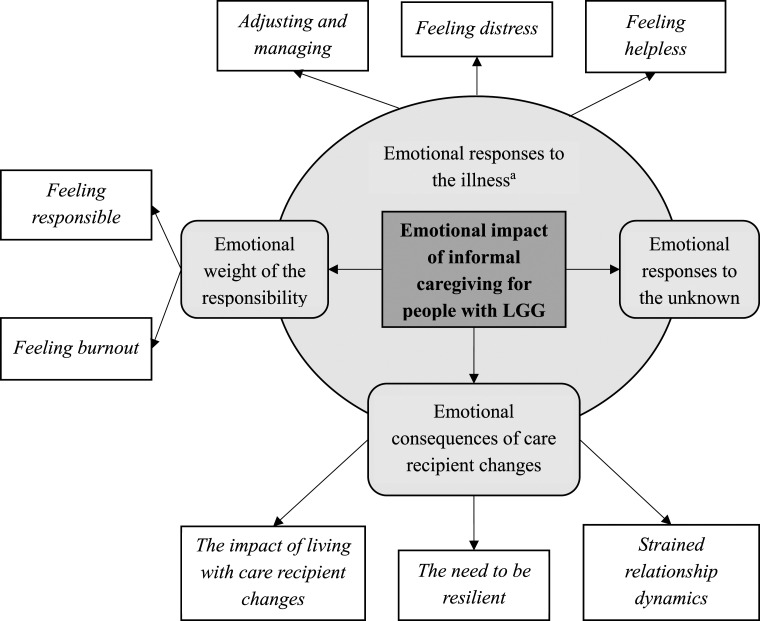
Overview of themes and subthemes for the emotional impact of informal caregiving for people with LGG. ^a^‘Emotional responses to the illness’ is an overarching theme that sets the context for the other themes.

Figure 1 provides an overview of the themes and associated subthemes; illustrative quotes are provided throughout the description of the themes below, with additional quotes in Table 1. Analysis resulted in one overarching theme, ‘*Emotional responses to the illness*’. This theme sets the context for the other three themes to express the emotional impact of being an IC for someone with an LGG, namely ‘*Emotional responses to the unknown*’, ‘*Emotional consequences of care recipient changes*’, and ‘*Emotional weight of the responsibility*’.

**Table 1.** Additional Supporting Quotes for All Themes and Subthemes, With Participant ID Number, Age at Interview, and Relationship to Care Recipient.

Theme	Subtheme	Illustrative quotes
1) Emotional responses to the illness	*Feeling helpless*	• ‘When you’re the mum, it’s supposed to happen to you, not your daughter. And you’re supposed to be able to kiss it better, and I couldn’t’. – IC3 (aged 78, mother)
		• ‘Whatever we did … she didn’t have a quality of life ... It was quite difficult for her’. – IC8 (aged 68, mother)
	*Feeling distress*	• ‘That is the big thing – you are constantly grieving for the life that you had. You are grieving for the life that you thought you were going to have, while still caring for this person who kind of still looks like your husband’. – IC1 (aged 38, wife)
		• ‘I did one to one counselling for a bit, for a chunk. I feel like I managed to get past the really low point but I know it’s there if I need it’. – IC18 (aged 48, wife)
		• ‘You’re not worried about anything, you’re only worried about her health and hopefully, like, that she’ll get back to some sort of normality’. – IC22 (aged 57, husband)
	*Adjusting and managing*	• ‘I think it’s very hard for the person who’s directly, like for me, it’s really hard for me to [provide care] day after day after day but you just do it because you love them and you want the best for them and you just get on with it’. – IC19 (aged 54, wife)
		• ‘You do almost block it out and not worry about it because you can’t spend your life worrying. You can’t spend years and years worrying. You have to live your life’. – IC6 (aged 50, sister)
2) Emotional responses to the unknown		• ‘It’s a bit like a rollercoaster because she’s having these six-monthly scans. So, once every six months you’re up on a high because it’s all clear and everything’s forgotten, tuck it to the back of your mind. And then all of the sudden you’re chewing your fingernails because she’s got a scan next week’. – IC3 (aged 78, mother)
		• ‘Something new is going to happen again, a new side effect because of the tumour and we have to re-adapt. I think subconsciously I’m always on the edge. I’m always expecting this to happen and I never fully rest’. – IC21 (aged 36, husband)
		• ‘I think [tumour progression] is constantly in your mind … It’s not something that’s happened, been dealt with, and it’s gone. You’re just waiting. And you spend your life waiting for it to come back’. – IC6 (aged 50, sister)
		• ‘It is like having a bit of a ticking time bomb because you’re not actually in a recovery stage. You’re not recovering from having the tumour and neither are you following an end pathway where you know where it’s going’. – IC12 (aged 66, wife)
3) Emotional consequences of care recipient changes	*The impact of living with care recipient changes*	• ‘She’d really have a go at me, really, really easily. So that was seriously difficult actually ... I’d say, “Okay fine, we’ll leave it,” and I’d back off and let it go … It was hard. It was difficult and a lot of times I was upset’. – IC8 (aged 68, mother)
		• ‘He’s took the dog for a walk and I’ve come home from work thinking, “Where on earth is he?” It’s the panic he’s given me … trying to explain to him, “You’ve left the keys on the outside of the door hanging. Anybody could have come past and burgled the place”’. – IC14 (aged 37, wife)
		• ‘It’s hard to tell always whether it’s hearing or memory because he pretends a lot, he makes up my answers a lot which is very frustrating’. – IC7 (aged 53, wife)
	*Strained relationship dynamics*	• ‘As far as I am concerned, my husband died on [date] … And I have been left with a complete stranger in my house’. – IC1 (aged 38, wife)
		• ‘I’ve taken on a lot of stuff, not that I’m trying to take his independence away. I don’t really trust him’. – IC14 (aged 37, wife)
		• ‘I would say that it [the diagnosis] has brought us closer together’. – IC22 (aged 57, husband)
	*The need to be resilient*	• ‘At the back of your mind you know you always have to be prepared. You feel like you have to stay mentally strong because any day now, or you don’t know when, you’ve got to be ready for what might happen if [care recipient] gets ill’. – IC12 (aged 66, wife)
		• ‘[care recipient] definitely went through a period of really not coping with family life and relationships and he would just go, just walk out the door and just disappear. The boys would get very upset about that. Rather than allowing myself time to be really annoyed that he was being really a bit crap, I was personally really upset. I had to shift the focus to the boys’. – IC18 (aged 48, wife)
		• ‘Sometimes we forget, myself and our daughters, forget that she was suffering with this and therefore were maybe not as tolerant with her periods of low mood or forgetfulness’. – IC13 (aged 51, husband)
4) Emotional weight of the responsibility	*Feeling responsible*	• ‘There is a greater weight of responsibility I feel rests on me now. I’m observing more. I’m aware a lot more. So when I use the word carer, ...a carer in the sense that I feel a greater weight of responsibility about how he is, where he is, is he okay, that rests on me’. – IC2 (aged 55, wife)
		• ‘I think it was, it’s just personal satisfaction to see that she is okay ... when someone’s unwell, you want to be fussing around them and helping them. But nothing needed doing. My brother-in-law done all [the housework] and my sister was just in bed asleep’. – IC6 (aged 50, sister)
		• ‘Again for a carer, I think it’s a lot of pressure if you cannot fund or financially look after the person that needs your support’. – IC21 (aged 36, husband)
	*Feeling burnout*	• ‘To deal with a bad mood and having to deal with irritability. It’s those types of things where you’re thinking, “I’d just love a week off,” you know ... but it’s not an option. I’m not going to get a week off every month just to have a break’. – IC1 (aged 38, wife)
		• ‘That caring aspect of it has taken its toll, yes. It’s taken its toll. I’ve never been a particularly emotional person but … I find myself getting close to tears quite easily which is unusual for me’. – IC24 (aged 67, husband)
		• ‘My work situation was quite stressful as well. So, you were trying to manage that and managing things at home. And I never really had problems, I would say, with my mental health but I was bursting out into tears for no good reason’. – IC15 (aged 44, wife)
		• ‘I was feeling very drained and exhausted from all the driving back and forth. Obviously it was emotional. I was trying to be strong for my daughter and be there for her. It was very difficult’. – IC8 (aged 68, mother)

### Emotional Responses to the Illness

This theme encompassed the feelings of distress and helplessness expressed by participants in response to the diagnosis, its impact on the care recipient, and potential prognosis; emotional adjustment was required to help participants manage.

#### Feeling Helpless

Feelings of helplessness were centred around participants feeling unable to improve the care recipient’s quality of life, due to the severity of the care recipient’s symptoms and impairments. This was often difficult to comprehend, especially for one mother, who wanted to be able to ‘*kiss it better*’. For many, helplessness was stimulated by the incurable nature of the diagnosis, as participants recognised they were unable to change the eventual outcome.

I just feel like I’ve had any hope taken away ... There’s no outcome from this other than [care recipient] dying and [care recipient] dying at a young age.

– IC14 (aged 37, wife)

#### Feeling Distress

Most participants felt substantial emotional distress following the care recipient’s diagnosis. Some spoke about feeling unprepared for the emotions that would ensue. Following initial feelings of anger and shock, subsequent care recipient changes (such as personality changes) meant some ICs reported ‘*grieving*’ the person they loved or the life they once had, or thought they were going to have. Several ICs also described a daily worry for the care recipient’s health and wellbeing, expressing concern for whether they would find a new sense of ‘*normality*’.

I wasn’t really prepared for the maelstrom of emotions that I experienced.

– IC23 (aged 56, husband)

#### Adjusting and Managing

Many participants spoke about the emotional adjustment they made to deal with the diagnosis and its consequences. This largely pertained to concerns about the potential prognosis. Some ICs attempted to remain optimistic and grateful and though difficult, to take each day as it comes. However, other strategies were maladaptive; some participants described a desire to avoid dwelling on the situation and deliberately suppressing their emotions, often acknowledging that such emotions were, therefore, not being appropriately processed. Some participants sought and received counselling to manage these feelings of loss and low mood.

It’s been, like, in a little box that I’ve got locked up and put somewhere that will get dealt with when it needs to get dealt with. So, probably not dealt with it.

– IC15 (aged 44, wife)

### Emotional Responses to the Unknown

This theme encompassed the feelings of fear, anxiety, panic, and uncertainty about the future, related to participants’ awareness that the tumour is/was incurable and likely to progress eventually. Though participants acknowledged that the tumour may remain stable for several months or years, for many ICs, the fear of progression was a constant feeling of knowing that something was going to happen at some point. This was exacerbated each time the care recipient experienced a symptom (e.g. headache and seizure), with some ICs feeling they were ‘*always on the edge*’.

It’s a constant black cloud hanging over you all the time, it’s something that is always there, you know it’s there, you can blank it out most of the time because it’s not having a direct impact … but you know that it’s not going away.

– IC13 (aged 51, husband)

Some participants specified timepoints when their anxiety intensified, referring particularly to the wait for scan results as ‘*no man’s land*’, as they wait to learn whether the tumour has progressed. Some ICs explained the impact of uncertainty on their ability to plan for their future. They felt restricted in how far they could plan in advance, and this had implications for their decision-making around both day-to-day issues (e.g. whether to book a holiday) and larger life plans (e.g. whether to have children).

It’s still that background nervousness of knowing how things can be, will be in the future. It’s perhaps not knowing but … my time horizon for planning and the future is now 6 months which is so much smaller than what it used to be.

– IC2 (aged 55, wife)

### Emotional Consequences of Care Recipient Changes

This theme encompassed the emotional consequences that participants felt in response to the care recipient’s cognitive and behavioural changes, due to the tumour and its treatment. These changes often had a profound impact on relationships, requiring ICs to be resilient.

#### The Impact of Living With Care Recipient Changes

Several participants described instances of the care recipient uncharacteristically displaying anger and irritability towards them, due to tumour-related changes in their personalities and ability to control their emotions. These situations were often stressful for the ICs, with the frequency and intensity of them leaving ICs feeling at the ‘*end of [their] tether*’. Low mood or negative attitudes exhibited by the care recipient influenced the mood of participants due, for example, to the care recipient not wanting to engage in enjoyable activities, such as day trips. Many participants expressed frustrations about the care recipient’s impaired memory, describing instances such as the care recipient forgetting important planned events; this frustration was most pronounced when the IC perceived that the memory problems influenced their safety (e.g. forgetting to lock the door).

The point at which I feel most down about it are the points [care recipient] is very down and stimies us doing any nice stuff.

– IC18 (aged 48, wife)

#### Strained Relationship Dynamics

Many participants spoke emotively about the impact that care recipient changes had on their relationship. Some ICs perceived that the care recipient had lost their identity; in severe cases, a few felt they were living with a ‘*complete stranger*’. A few participants, however, expressed how the diagnosis made the relationship stronger. Only a very few participants reported open communication with the care recipient about the illness; in contrast, most spoke about the care recipient deliberately suppressing the topic. This limited whether ICs could voice their concerns, often leading to the ICs suppressing their emotions, dealing with them alone and internally.

He told me not to talk about it. He told me not to cry about it. We didn’t talk about it for years, literally years unless he actually had an appointment. Then about five years ago I was training and [my emotions] all came out. I came home and said, ‘We need to talk about this’.

– IC7 (aged 53, wife)

Several participants described a shift in the balance of household responsibilities (e.g. childcare and housework), describing how they had taken on more because of the care recipient experiencing fatigue or because of a lack of trust in the care recipient due to memory problems. For some, this added an emotional strain to their relationship with the care recipient, with one IC stating that they started to ‘*resent*’ their partner.

#### The Need to Be Resilient

Several participants expressed the need to be resilient in response to care recipient changes, in particular the need to maintain control over their own emotions. This was manifested in numerous ways. Some ICs perceived a need to be mentally strong for when the care recipient’s illness progressed. Other participants detailed the need for patience and communication to avoid further aggravating the care recipient. For instances where the care recipient was not managing well and behaving poorly within the family, ICs spoke about having to prioritise their children’s wellbeing over their own emotions. Over time, tolerance of care recipient changes reduced for some, as ICs sometimes forgot about the illness and its consequences in times of ‘normality’.

I think the key to [living a new way] is communicating so that she doesn’t feel less of a person, she doesn’t feel frustrated and I don’t feel that I’m having to do everything or that I’m resentful because she’s maybe snappy because she’s fatigued.

– IC23 (aged 56, husband)

### Emotional Weight of the Responsibility

The pressure and demand of the associated responsibilities of being an IC for someone with an LGG meant that they often experienced emotional exhaustion.

#### Feeling Responsible

Many participants felt a weight of responsibility in general for the care recipient’s wellbeing (more so than before diagnosis), as well as specific pressures of being able to provide necessary financial and practical (e.g. medication reminders) support. This pressure was exacerbated if the IC had issues with their own health, raising concerns over who would fulfil the caregiving responsibilities. For non-spousal ICs, there was an emotional conflict of a perceived need to assume more responsibilities that were already being fulfilled by the care recipient’s spouse, for example, helping with the housework.

I was always concerned, “What happens if I get ill?” because I’ve got lots of health problems myself. What was going to happen?

– IC8 (aged 68, mother)

#### Feeling Burnout

Several participants spoke emotively about feeling ‘*drained*’ and ‘*exhausted*’ as a result of attempts to fulfil what they perceived were their caregiving responsibilities; they described how the tumour was ‘*taking its toll*’. Some ICs described how it was too much to manage the combined stress of work and caregiving; for ICs living apart from the care recipient, trying to be there as often as possible felt demanding. Overall, a lack of respite from the perceived caregiving responsibilities and living with care recipient changes had a substantial impact on emotional exhaustion, with potentially detrimental consequences; for example, one IC described a desired to leave and not come back, saying:

One day last week I just felt like getting in the car and driving off and just not coming back because it just all got on top of me.

– IC19 (aged 54, wife)

## Discussion

The tumour- and treatment-related symptoms and impairments experienced by people with LGGs mean those closest to them, particularly partners, often adopt a caregiving role. This study outlines how ICs of people with LGGs experience substantial emotional impact due to caregiving demands, the uncertain prognosis, and living with care recipient changes and their consequences. Such impact may be sustained for long periods due to the potential for a long-term prognosis. While ICs try to adjust to the situation or be resilient to manage, these issues coupled with the perceived weight of responsibility result in some experiencing emotional exhaustion.

Feelings of helplessness following a brain tumour diagnosis in our study resonate with findings for ICs of people with HGGs (Wideheim et al., 2002); we expand on this to show that these feelings can be long lasting through the disease course with some ICs feeling helpless because they were unable to offer their care recipient a good quality of life. These feelings exacerbated feelings of anxiety and distress and intensified still when the care recipient exhibited low mood (Northouse et al., 2012).

The incurable nature of LGGs meant our participants often described being in what could be considered a liminal state (Sabo, 2014), grieving a life they once had, with future plans disrupted by an undeterminable wait for an inevitable outcome; this presented what appeared to be the greatest source of emotional distress for ICs of people with LGGs. The impact and potential support needs related to the fear and uncertainty of ‘when’ the tumour will progress (Amaresha et al., 2015) are distinct from the fear of ‘if’ the cancer will recur, experienced by ICs of people with cancers that have more favourable prognoses (Smith et al., 2021).

Our findings are congruent with the conceptual model that the neurological (i.e. cognitive and neuropsychiatric) status of the person with a brain tumour can influence the level of emotional distress felt by the IC (Sherwood et al., 2004). We expand on qualitative work in ICs of people with brain tumours, which acknowledges the challenges of living with care recipient changes (e.g. personality changes and memory deficits) (Schubart et al., 2008), to underline the emotional impact associated with these challenges. Our findings indicate that care recipient changes often placed strain on the relationship dynamic – in part due to issues with trust – with consequent imbalances (e.g. with housework and childcare) exacerbating caregiving burden. Hence, it is perhaps unsurprising that we found several reports of burnout in ICs.

Our findings highlight the role of adjustment (e.g. acceptance and negotiating changes in family roles) (Cavers et al., 2013) and resilience (e.g. tolerance of care recipient changes) (Sun et al., 2021; van Roij et al., 2021) as protective factors to reduce the emotional impact of being an IC for someone with an LGG. However, in our findings, adjustment also encompassed avoidance, whereby the IC suppressed their worries, particularly regarding fear of tumour progression. This can be maladaptive (Baumstarck et al., 2018) and may be exacerbated by the reluctance of the care recipient to communicate about the illness, which was observed here and has been reported in a past study of how spouses deal with a glioma diagnosis (Salander & Spetz, 2002). Therefore, we underline the importance of identifying or providing avenues of emotional support for ICs of people with LGGs. We have reported elsewhere from this dataset that such support may come from informal networks (e.g. close friends), where available (Murrell et al., 2023). As has been reported elsewhere, in dyads affected by cancer, the needs of the care recipient often take primary focus (Cornwell et al., 2012; Sterckx et al., 2013), meaning ICs might not obtain support for themselves. Indeed, our data included few reports of emotional support being sought or received by participants, often citing poor awareness of available support for ICs.

Overall, this study extends a limited evidence base, shedding new light on the potential emotional support needs of ICs of people with LGGs. By exploring emotional impacts in depth and detail, we go beyond past work, which reported presence of emotional distress in ICs of people with LGGs (Edvardsson & Ahlström, 2008). Specifically, we have advanced understanding of the emotional impact of caregiving responsibilities in LGGs, living with care recipient changes, and the protective factors that helped ICs to manage such impact. By identifying (currently unmet) needs for emotional support, and what may be driving these, our findings can be viewed as the first step in developing solutions to address emotional distress among this group.

### Implications

Our results emphasise the importance of advice and signposting to ensure awareness of, and access to, available emotional support for ICs. Particularly, ICs of people with LGGs are navigating a distinct level of fear and future uncertainty, due to the incurable – albeit often longer term – nature of the condition. We also found that ICs can be emotionally impacted by, and experience challenges with adjustment to, assuming caregiving responsibilities, living with care recipient changes, and shifts in family dynamic. Thus, ICs of people with LGGs may benefit from resources that highlight what they might expect (e.g. care recipient personality changes) and provide advice or (ideally) active strategies that promote emotional adjustment and equip ICs with the skills to develop positive coping mechanisms. These would all need careful testing to make sure they do not exacerbate anxiety. Ultimately, ICs’ potential support needs should be monitored alongside patient needs assessments so that they are offered the support and information required to ensure they can sustain their responsibilities as an IC. This is important because the wellbeing of the care recipient can be negatively affected if the IC has unmet needs (Hodgkinson et al., 2007).

### Strengths and Limitations

Our qualitative approach allowed participants to explain how *they* experience the emotional impact of being an IC for someone with an LGG. Since all participants spoke very strongly about the emotional impact and their needs for emotional support, the present analysis focused on that one dimension of the interviews and data. This does not mean that ICs also do not have other support needs that are important to them, and these should not be disregarded. We are confident that reasonable data sufficiency was achieved in this analysis, with sufficient data to support and understand the emotional impact experienced by ICs of people with an LGG.

Partial recruitment through the Brain Tumour Charity’s networks, as a consequence of COVID-19, means the possibility cannot be discounted that some participants were self-selected opting to participate because they were more ‘active’ in their caregiving role and/or had more time and interest to participate in the study. Our participants were largely spousal ICs, so findings may overemphasise impacts on spousal relationships and the findings may not fully generalise to other ‘types’ of ICs (e.g. other family members). There was a sex imbalance in our dataset; while males are commonly underrepresented in brain tumour caregiving literature (Chen et al., 2021), it is possible that female ICs experience greater levels of caregiving burden, anxiety, and depression (Choi et al., 2012; Q. P. Li et al., 2013) or are more willing to be open about their emotional experiences due to social and cultural roles. We lacked information on the time since the care recipient’s diagnosis and that is a limitation.

Finally, while this analysis focused entirely on the perspective of the caregiver, it is important to recognise that caregiving occurs within the context of a relationship between the caregiver and the care recipient; the care recipient may also provide support to the so-called caregiver, and the balance of this ‘giver–receiver’ relationship may be fluid and change over time. Though we interviewed people with LGGs in another phase of the Ways Ahead project, these were generally not the care recipients of the interviewed ICs. Moreover, in our interviews, we did not probe specifically about any support the IC might have received from the care recipient. Future research among dyads affected by LGGs, ideally with a longitudinal design, would be of value to explore the dynamic nature of this relationship.

## Conclusions

The emotional impact encompassed ICs’ responses to the illness, uncertain prognosis, consequences of care recipient changes, and the toll of assuming a caregiving role. Emotional impact in one area frequently exacerbated the impact in another, and often vice versa; for example, the impact of living with care recipient changes contributed to feeling burnout, while those feeling burnout found it more difficult to live with care recipient changes. We highlight how adjustment and resilience can be influential in alleviating the emotional impact. Further consideration of ways to identify and fulfil the emotional support needs of ICs of people with LGGs is required; this has potential to benefit both the ICs and their care recipients.

## Supplemental Material

Supplemental Material - “A Constant Black Cloud” the Emotional Impact of Informal Caregiving for Someone With a Lower-Grade Glioma Click here for additional data file. Supplemental Material for ‘A Constant Black Cloud’: The Emotional Impact of Informal Caregiving for Someone With a Lower-Grade Glioma by Ben Rimmer, Michelle Balla, Lizzie Dutton, Joanne Lewis, Richéal Burns, Pamela Gallagher, Sophie Williams, Vera Araujo-Soares, Tracy Finch, and Linda Sharp in Qualitative Health Research
