# Supplementary material for: ‘A Constant Black Cloud’: The Emotional Impact of Informal Caregiving for Someone With a Lower-Grade Glioma
Source: Qual Health Res. 2023 Nov 15;34(3):227–38. doi: 10.1177/10497323231204740 (PMC10768339; doi:10.1177/10497323231204740)
Supplement: Supplemental Material - “A Constant Black Cloud” the Emotional Impact of Informal Caregiving for Someone With a Lower-Grade Glioma [file sj-pdf-1-qhr-10.1177_10497323231204740.pdf]

Topic guide for family-members & friends interviews

The direction and content of the interview, the order in which topics are covered, and the precise wording of questions and probes, will be determined by the issues and topics raised by, and the personal circumstances and experiences of, the interviewee. This topic guide therefore functions as an issue checklist for the interviewer.

Introductory questions:

Would you like to start by telling me a bit about yourself?
Can you tell me a bit about your relationship with [care recipient]? When was [care recipient] diagnosed with a brain tumour? What was that like?

Topics to cover:

- Experiences of supporting someone living with a brain tumour and perceived experiences of the care recipient*
    - Transition from treatment
    - Physical impact
    - Psychological impact
        - [Care recipient] personality changes
    - Cognitive impact
    - Emotions relating to brain tumour and its recurrence
    - Relationships, parenting & family roles
        - Interviewee's own support network
            - Influence on capacity to provide care
    - Work
    - Transport
    - Hobbies and interests
    - Finances
    - Managing medications and health appointments
    - Seeking support
    - Healthcare support
    - Coping and self-efficacy
- Most important aspect of life impacted for interviewee and care recipient
- Difficulties of living with and supporting an individual with a brain tumour
    - What they find has worked in their provision of support
- Desired support and intervention design preferences
    - Role of family and friends in supporting people with brain tumours

**For each topic, cover the following:*

- What the impact was
    - What impact this had on interviewee, where applicable
- How it was managed
- Support provided by the interviewee
- What support was received (for interviewee and care recipient)
- What support was needed (for interviewee and care recipient)
- When it was most impacted
- When the support was needed

Closing questions:

Is there anything you would like to tell me that we haven't already discussed?
Do you have any questions for me?
